# Awareness, Prevention, and Risk Factors of Non-Pigmented Skin Cancers: A Cross-Sectional Study Among Patients Undergoing Surgical Treatment

**DOI:** 10.3390/jcm14186512

**Published:** 2025-09-16

**Authors:** Monika Wojarska, Paulina Bernecka, Jerzy Jankau

**Affiliations:** 1 Plastic Surgery Department, University Clinical Center in Gdańsk, Mariana Smoluchowskiego 17 80-110, 80-211 Gdansk, Poland; mlacka@gumed.edu.pl (M.W.); jjankau@gumed.edu.pl (J.J.); 2 Faculty of Medicine, Medical University of Gdańsk, Marii Skłodowskiej-Curie 3a, 80-210 Gdansk, Poland

**Keywords:** skin cancer, skin cancer early detection and prevention, surgery, social issues

## Abstract

**Background/Objectives**: Skin cancer is the most common cancer worldwide, creating a significant burden on healthcare systems. According to the World Health Organization, 1.5 million new cases are reported annually, though the actual number is likely higher due to underreporting. The main risk factor is UV radiation, with additional contributors such as smoking, older age, and outdoor work. Basal cell carcinoma (70–80%) and squamous cell carcinoma are the most prevalent non-pigmented skin cancers. This study assessed the knowledge of patients undergoing surgical treatment for facial skin cancer regarding risk factors, prevention, treatment, and health-related behaviors. The goal was to guide educational strategies aimed at reducing disease incidence and improving outcomes. **Methods**: A cross-sectional study of 220 patients treated at the UCK Department of Plastic Surgery (April–August 2024) was conducted. Participants completed anonymous questionnaires on demographics, medical history, beliefs about lesions, and sun-protective behaviors. Clinical data included tumor location, size, histopathology, and excision completeness. Statistical significance was set at *p* < 0.05. **Results**: Patients were on average 71 years old; 61% had a secondary education. Sun protection habits varied by education and gender. SPF use was higher among those with higher education (79.55%) and among women (55.83%). SPF users had smaller lesion diameters (*p* < 0.001). However, 71% delayed seeking care for over a year, often due to misperceptions. **Conclusions**: There is a notable lack of awareness about skin cancer, especially prevention and early detection. Education and gender influence protective behaviors. Tailored educational initiatives may help reduce incidence and promote earlier diagnosis.

## 1. Introduction

Skin cancers are the most common cancers in the world, posing a major challenge to the healthcare system [1,2]. According to the World Health Organization, 1.5 million new cancer diagnoses are made each year. At the same time, the exact number of diagnoses remains underestimated due to a lack of proper reporting of the disease [3,4,5].

The most well-documented risk factor for developing skin cancer is increased exposure to UV radiation [2,6]. Other risk factors include cigarette smoking, older age, male gender, history of skin cancer, skin burns, fair complexion, use of tanning beds, and jobs involving being outdoors, such as gardeners [4,7].

The most common non-pigmented skin cancer is basal cell carcinoma, which accounts for about 70–80% of all diagnosed non-pigmented skin cancers. The second most common cancer is squamous cell carcinoma. These cancers are not only a health problem, but also contribute to a significant financial burden on healthcare systems [8].

Studies show that patients suffering from skin cancer have a higher risk of skin cancer recurrence [9].

There is not much known about illness perception in patients with skin tumors [10,11,12]. For this reason, it is important to conduct research on the level of awareness of these patients about prevention, the causes of cancer, and the presence of risk factors.

By understanding how patients perceive these aspects, we can better tailor educational interventions that can effectively reduce the incidence of skin cancer and improve patients’ overall quality of life.

The purpose of our study is to analyze the knowledge of patients undergoing surgical treatment for facial skin cancer about risk factors for the disease, treatment, prevention, and health-promoting behaviors.

## 2. Materials and Methods

Study design and study population: a cross-sectional study was conducted among patients of the UCK Department of Plastic Surgery who underwent surgical treatment of skin cancer between April and August 2024. The study enrolled 220 patients with a histopathologically confirmed diagnosis of skin cancer who consented to participate in the study and completed an anonymous questionnaire.

Research tools: The survey consisted of a series of closed-ended questions regarding demographics (age, gender, education level), medical history (previous sunburn, family history of skin cancer), duration of observation of the cancerous lesion, beliefs about the causes of the lesion, treatment methods used, occupational sun exposure (such as construction workers, bricklayers, plasterers, gardeners, farmers, foresters, etc.), smoking, and use of sunscreen (mechanical and chemical). Although the Fitzpatrick skin phototype assessment was initially included in the questionnaire, we decided not to report it in the study results. The examined population in Poland consisted predominantly of patients with phototype I. Moreover, due to the advanced age of participants, the vast majority had gray hair, which further reduced the accuracy of the assessment and the clinical applicability of the Fitzpatrick classification in this cohort. Data collection: Surveys were conducted with patients during hospital admission. Patients completed the surveys independently. Research staff were available during survey completion to provide support and clarification of question content, and to confirm complete survey completion.

Clinical parameters: The location and size of the tumor lesions in millimeters, histopathological examination result, the lesion’s excision completeness, and any follow-up treatment were assessed as part of the subject examination to further correlate the clinical data with the survey responses.

Data analysis: Data were statistically analyzed using IBM SPSS Statistics software, version 25 (IBM Corp., 1 New Orchard Road, Armonk, NY, USA). Descriptive statistics were used to show the demographic characteristics of the participants and their responses to the survey questions. The Chi-square test was used to assess associations between categorical variables, such as gender and type of prevention used. The level of statistical significance was set at *p* < 0.05. Fisher’s exact test was applied for each pair of lesion locations to assess whether there was a statistically significant association between lesion location and complete excision, as well as between cancer type and whether the individuals worked outdoors or had a history of sunburn. The Kruskal–Wallis test was performed to compare medians between groups. A *p*-value (shown in the last column) lower than α = 0.05 indicated a statistically significant difference between at least one pair of medians. In the next step, a post hoc test was conducted to identify which specific pairs of groups differed significantly.

## 3. Results

### 3.1. Patient Characteristics

#### 3.1.1. Demographics

##### Gender, Age

220 patients surgically treated for skin cancers in the head and neck were included in the study. There were 120 women and 100 men, ranging in age from 18 to 97 years. The average age of patients in the study group was 71.17 years.

##### Education

61% of patients had secondary education, higher education 20% of patients had higher education, and 19% of patients had primary education.

##### Characteristics of the Work Performed

17% of patients had jobs that involved being outside all the time.

##### Medical History

Sunburn: 36% of patients had a history of skin burns, while 64% had not experienced such burns.

##### History of Skin Cancer

76% of patients had no history of skin cancer other than the one causing hospitalization; 24% of patients had a history of skin cancer.

91% of patients had no family history of skin cancer, and 9% of patients confirmed a family history of skin cancer.

### 3.2. Perception of the Disease and Treatment

Causes of skin lesions: 19% of patients believed their skin lesion was caused by acne, 12% by dry skin, 9.5% by mechanical trauma, and 4.5% by a local skin infection. 55% did not associate the lesion with any of the above factors.

#### 3.2.1. Treatment

Self-treatment: 25% attempted to treat the lesion on their own before consulting a doctor, while 75% did not take any treatment action before visiting a doctor.

Treatment decisions: 51% of patients consulted a doctor after being persuaded by relatives, while 49% decided on their own.

#### 3.2.2. Sun Exposure and Protective Behavior

SPF use: 47% of patients used SPF cream, mostly on sunny days (80%) or only in summer (86%).

89% of patients had never used a tanning bed, and 11% had used one in the past.

Other methods of protection (mechanical): 55% wore baseball caps, 55% used sunglasses, 30% wore long-sleeved clothing, and 60% avoided sun exposure during peak sunlight hours.

### 3.3. Characteristics of the Lesion

74% of the lesions removed by histopathological examination were malignant lesions, 20% were benign, while 6% were precancerous lesions.

In the histopathological diagnosis, 56% of the lesions were basal cell carcinoma of the skin, 14% squamous cell carcinoma, 7.7% other benign lesions, 6.4% keratosis precancerosa, 5% were seborrheic warts, 3.6% benign pigmented lesions, 3.2% hemangiomas, 2.3% melanoma, and 1.4% other malignant lesions.

There was no correlation between tumor type and a history of skin burns, nicotinism, or working outside (*p* > 0.05).

87% of lesions on histopathology were excised completely according to oncology guidelines.

Excision completeness was not statistically significantly related to lesion diameter or lesion location (*p* > 0.05).

### 3.4. Location of the Lesion

In 29.8% of cases, the lesion was located on the nose, 19% on the upper or lower eyelid, 13% on the cheek, 9.5% of lesions were located on the forehead, 8.6% involved the lips, 6.4% were located on the auricle, 5.5% on the temporal region, 5% of lesions were located outside the face and 3.2% in the parietal region.

Lesion size ranged from 2 mm to 80 mm and averaged 14.77 mm with a median (min-max) of 12

### 3.5. Duration of Observation of the Lesion

71% of patients observed their lesion for more than a year before they decided to seek medical advice, 13% observed their lesion for 7–12 months, 10% observed their lesion for 4–6 months, and 5.9% of patients declared that they had observed their lesion for 1–3 months before their medical visit.

There was no correlation between the time of observing the change and education and gender (*p* > 0.05).

### 3.6. SPF

In total, 47% of patients declared that they use SPF, including 79% of patients with higher education, 45.93% of patients with secondary education, and 17.07% of patients with primary education.

It was shown that there is a statistically significant relationship between education and SPF use (*p* < 0.001).

The percentage of SPF users is statistically significantly higher in the group of patients with secondary education compared to those with primary education (45.93% vs. 17.07%, *p* < 0.01).

The percentage of SPF users is statistically significantly higher in the group of people with higher education compared to those with primary education (79.55% vs. 17.07%, *p* < 0.001).

The percentage of SPF users is statistically significantly higher in the group of those with higher education compared to those with secondary education (79.55% vs. 45.93%, *p* < 0.001).

**Figure 1:**
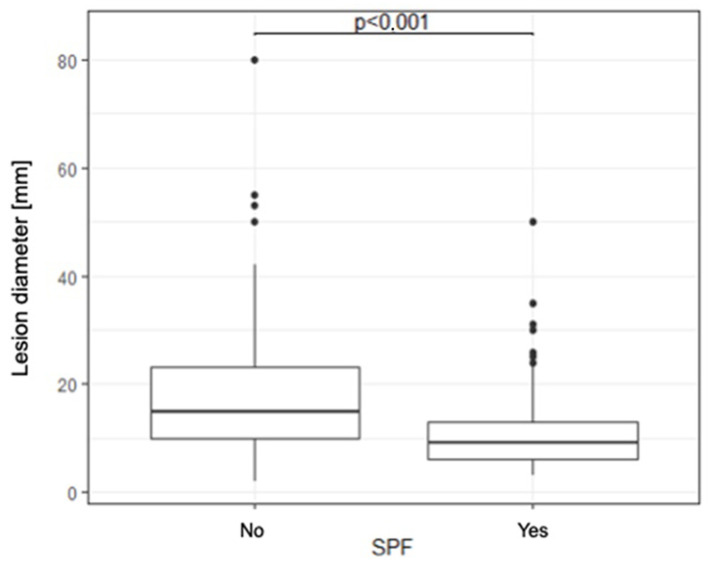
A box plot illustrating the relationship between the diameter of the lesion and whether the patient used SPF.

The median lesion diameter in SPF users is statistically significantly smaller compared to the group that does not use SPF (15 [10–23] vs. 9 [6–13], *p* < 0.001) (Figure 1).

Women were found to use SPF more often than men (55.83% vs. 37%, *p* < 0.01).

### 3.7. Lesion Diameter

**Figure 2:**
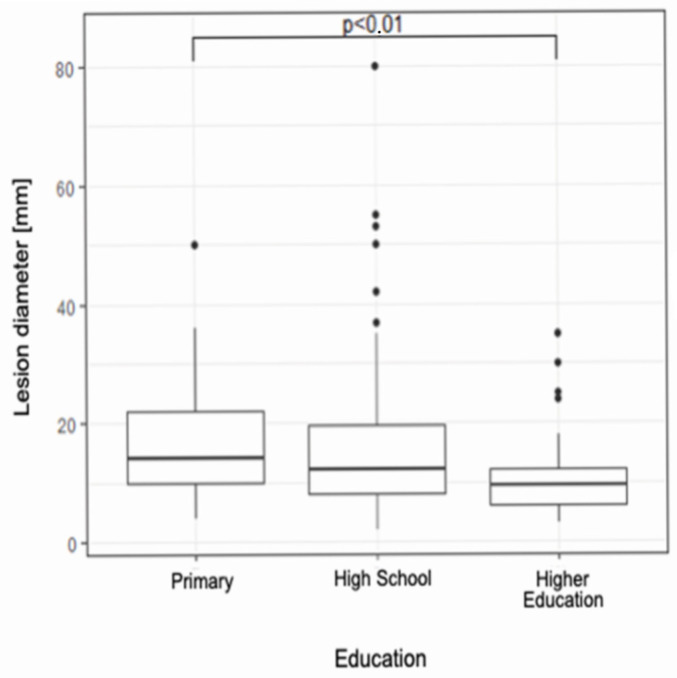
A box plot illustrating the dependence of education level on lesion diameter.

The median lesion diameter in the group with tertiary education is statistically significantly smaller compared to the group with primary education (10 [6–12] vs. 14 [10–22], *p* < 0.01) (Figure 2).

**Figure 3:**
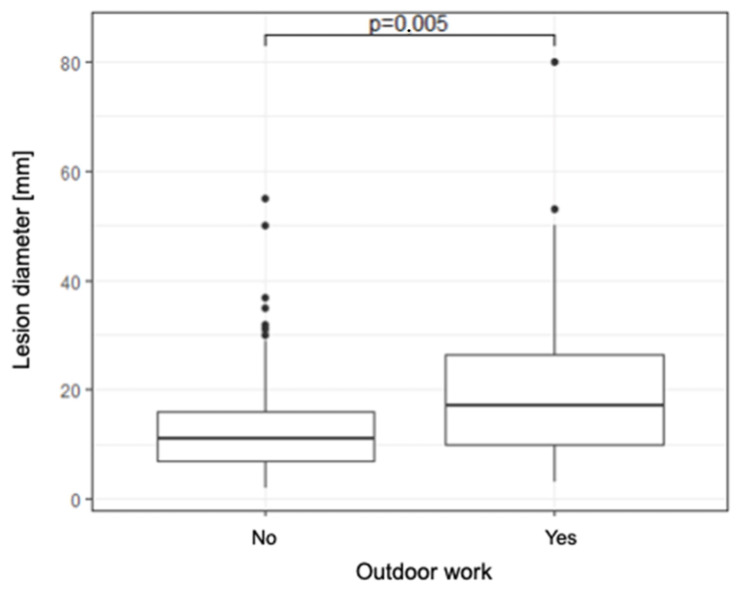
A box plot illustrating the relationship between outdoor work and lesion diameter.

The median diameter of the lesion in those who work outside is statistically significantly larger compared to those who do not work outside (17 [10–27] vs. 11 [7–16], *p* < 0.01) (Figure 3).

However, there was no correlation between median lesion diameter and smoking or medical history of burns (*p* > 0.05).

## 4. Discussion

The questionnaire was collected at the time of admission to the hospital for surgical treatment for facial skin cancer. This means that the diagnosis of cancer had no effect on previous photoprotection behavior. Only 47% of patients declared that they used sunscreen, of which 80% used it only in sunny weather, while 86% used it only in summer. The correct rules of photoprotection call for daily application of sunscreen regardless of the weather and reapplication during the day.

Unfortunately, a very large proportion of patients not only did not apply the cream, but also did not know what it was. Indeed, women were more likely to apply sunscreen, which is consistent with the literature indicating that women are more likely than men to engage in health-promoting behaviors, especially in the context of sun protection [11,13]. Older patients were much more likely to avoid leaving the house during hours of high sunlight due to the oppressiveness of the heat, unknowingly using this type of photoprotection. At the same time, a very large proportion of these patients detailed that they liked to be heavily tanned in their youth. This may mean that lack of photoprotection in youth is a risk factor, and behaviors preferred in older life can no longer reverse carcinogenesis [13]. In turn, the authors indicate that avoiding sun exposure is more effective than using sunscreen alone because patients protected in this way consciously prolonged their time in the sun [14,15]. This stands in line with the results of the survey, where patients declared that they use sunscreen only on vacation. At the same time, an interesting observation is the relationship between the level of education and the use of SPF—the higher the level of education, the higher the percentage of patients declaring that they use sunscreen. This is most likely due to increased awareness of the etiology of skin cancer formation [16,17]. UV radiation is the most common and best identified risk factor for skin cancer.

A correlation between SPF use and lesion diameter was also noted among patients. Patients who reported sunscreen use presented with lesions of significantly smaller diameter. Smaller lesions are typically associated with lower local disease advancement, which makes them easier to treat and is linked to a more favorable prognosis. SPF protects against sunlight (both UVA and UVB radiation), and sun exposure is the best-documented risk factor for skin cancers. Regular use of sunscreen may contribute to delayed tumor development or slower progression; therefore, the lower number of skin cancers observed in patients using SPF may indicate that these individuals have greater awareness of the risks associated with skin neoplasms and are more likely to seek medical attention promptly.

These findings carry important public health implications. Larger lesions typically require more extensive surgical procedures and, in some cases, general anesthesia. When located on the face, excision of large skin cancers often necessitates more extensive surgical interventions and frequently involves multi-stage reconstructive treatment, which is highly demanding for patients and generates substantial costs for the healthcare system. Promoting consistent sunscreen use may therefore contribute not only to reducing the incidence of skin cancer but also to lowering the severity of surgical management and its economic impact.

At the same time, given that the higher the level of education, the lower the median diameter of the cancerous lesion, it is difficult not to combine the two facts above. Patients with higher education showed greater awareness of skin cancer, its prevention, and clinical picture- they were quicker than the other groups of patients to correctly recognize the disease process in themselves and report it to the doctor.

Smoking and burns are well-established risk factors for the development of both squamous cell carcinoma (SCC) and basal cell carcinoma (BCC). In the present analysis, Fisher’s exact test was applied to assess the association between tumor type and smoking status or sunburns.

The obtained *p*-value did not demonstrate a statistically significant correlation. The lack of statistical significance regarding the association between risk factors such as smoking or sunburns and tumor type in our analysis may be explained by the relatively small sample size, which limited the statistical power, thereby reducing the ability to detect subtle associations.

Almost half of the patients included in the study (45%) had misconceptions about the etiology and nature of the neoplastic lesion causing hospitalization [18]. Denial and lack of knowledge about the typical course or clinical picture of skin cancer can be a reason for delaying medical consultation and the start of treatment [19].

This is supported by the fact that as many as 71% of patients observed a change in themselves for more than a year before they decided to seek medical advice, and an additional 49% of patients did so only at the advice of someone close to them.

## 5. Study Limitations

1. Single-center study: The research was conducted at a single facility (UCK Department of Plastic Surgery), limiting the generalizability of the findings to other populations and regions.
2. Limited demographic diversity: The majority of participants were older adults (average age of 71 years), which may not reflect the experiences of younger populations at risk of skin cancer.
3. The lack of prior regional data: One limitation of this study is the lack of prior regional data on patient awareness of non-pigmented skin cancers, which limits direct comparison. Nonetheless, this gap emphasizes the novelty and relevance of our research and the need for further studies in this area.
4. The lack of data on skin phototypes (Fitzpatrick scale): Data on participants’ skin phototypes according to the Fitzpatrick scale were not collected, which may influence susceptibility to skin cancer and affect the generalizability of the findings.
5. Potential recall bias in self-reported behaviors: The study relied on self-reported behaviors, which introduces the potential for recall bias. Participants may have over- or under-reported sun-protective behaviors or other relevant exposures, which could influence the observed associations.

In conclusion, while the study provides valuable insights, these limitations highlight the need for more diverse, multi-center, and long-term studies to fully understand the complexities of skin cancer prevention and treatment.

These findings underscore the need to intensify educational efforts targeting various segments of the population about the importance of sun protection and early diagnosis of skin cancer. As the results of the study indicate, strengthening awareness about the importance of prevention and early intervention can significantly improve health outcomes in the population.

It would be highly beneficial to establish educational initiatives specifically targeting non-pigmented skin cancers, as public awareness of these malignancies remains limited compared to melanoma. These actions should emphasize recognition of early warning signs, risk factor modification, and the importance of regular dermatological examinations, thereby addressing existing gaps in patient knowledge and awareness.

The study confirms that education level and gender have a significant impact on photoprotective behavior. It recommends implementing targeted educational programs that can effectively raise awareness and change health behaviors to reduce the risk of developing skin cancer.

## Data Availability

The data presented in the article cannot be made publicly available due to the need to protect the anonymity of the study participants. The responses provided in the completed questionnaires are private and confidential, and disclosing them could violate research ethics and data protection regulations. Therefore, full access to the data is restricted to individuals directly involved in the analysis or, upon reasonable request, in accordance with established procedures and the participants’ consent.
